# Engaging community-dwelling older adults in fall prevention programs: a qualitative study on strategies promoting participation in fall prevention programs among community-dwelling older adults

**DOI:** 10.3389/fpubh.2023.1150659

**Published:** 2023-06-30

**Authors:** Meike van Scherpenseel, Lidia van Veenendaal, Lennie Donné, Saskia te Velde, Amber Ronteltap

**Affiliations:** ^1^Research Group Innovation of Human Movement Care, Research Center for Healthy and Sustainable Living, HU University of Applied Sciences Utrecht, Utrecht, Netherlands; ^2^Research Group Proactive Care for Older Adult People Living at Home, Research Center for Healthy and Sustainable Living, HU University of Applied Sciences Utrecht, Utrecht, Netherlands; ^3^Bachelor Nursing Studies, Institute for Nursing Studies, HU University of Applied Sciences Utrecht, Utrecht, Netherlands; ^4^Research Group Innovation in Healthcare Processes in Pharmacology, Research Center for Healthy and Sustainable Living, HU University of Applied Sciences Utrecht, Utrecht, Netherlands; ^5^Program Group Persuasive Communication, Department of Communication Science, University of Amsterdam, Amsterdam, Netherlands

**Keywords:** fall prevention programs, community-dwelling older adults, strategies, participation, implementation

## Abstract

**Introduction:**

Fall rates and fall-related injuries among community-dwelling older adults (≥65 years) are expected to increase rapidly, due to the aging population worldwide. Fall prevention programs (FPPs), consisting of strength and balance exercises, have been proven effective in reducing fall rates among older adults. However, these FPPs have not reached their full potential as most programs are under-enrolled. Therefore, this study aims to identify promising strategies that promote participation in FPPs among community-dwelling older adults.

**Methods:**

This is an exploratory qualitative study. Previously, barriers and facilitators for participation in FPPs by older adults had been identified. Next, six strategies had been designed using the Intervention Mapping approach: (1) reframing; (2) informing about benefits; (3) raising awareness of risks; (4) involving social environment; (5) offering tailored intervention; (6) arranging practicalities. Strategies were validated during semi-structured interviews with community-dwelling older adults (*n* = 12) at risk of falling. Interviews were audio-recorded, transcribed, and analyzed following a qualitative thematic methodology, with a hybrid approach.

**Results:**

All strategies were considered important by at least some of the respondents. However, two strategies stood out: (1) reframing ‘aging’ and ‘fall prevention’: respondents preferred to be approached differently, taking a ‘life course’ perspective about falls, and avoiding confronting words; and (2) ‘informing about benefits’ (e.g., ‘living independently for longer’); which was mentioned to improve the understanding of the relevance of participating in FPPs. Other strategies were considered important to take into account too, but opinions varied more strongly.

**Discussion:**

This study provides insight into potential strategies to stimulate older adults to participate in FPPs. Results suggest that reframing ‘aging’ and ‘fall prevention’ may facilitate the dialogue about fall prevention, by communicating differently about the topic, for example ‘staying fit and healthy’, while focusing on the benefits of participating in FPPs. Gaining insight into the strategies’ effectiveness and working mechanisms is an area for future research. This could lead to practical recommendations and help professionals to enhance older adults’ participation in FPPs. Currently, the strategies are further developed to be applied and evaluated for effectiveness in multiple field labs in a central Dutch region (Utrecht).

## 1. Introduction

As the worldwide population ages, falls and falls-related injuries among community-dwelling older adults over 65 years of age are increasing rapidly (1, 2). It has been estimated that approximately one-third of the community-dwelling older adults aged 65 years and older, fall at least once a year, making it an important global issue (3, 4). Falling can have serious consequences for older adults, such as pain, loss of confidence, loss of independence, lower quality of life, and even death (5). Besides personal distress, costs associated with (non-)fatal injuries are high and increasing substantially (6). Therefore, preventing falls is of utmost importance to halt this upward trend (2).

In general, physical activity has been related to numerous physical and mental health benefits and well-being, such as the decreased risk of developing stroke, lower blood pressure, and lower risk of cognitive decline and all-cause mortality (7, 8). Several studies have also found that exercise prevents falls and fractures in older people (9, 10). Moreover, in fall prevention, other factors besides exercise, need to be addressed. To date, several fall prevention interventions, targeting modifiable risk factors, have been proven effective in reducing the rate of falls among community-dwelling older adults (11, 12). These interventions differ in focus, depending on the risk profile of the individual, and are recommended to include multiple components (12). These multicomponent interventions may consist of medication reviews, environmental modification, and interventions for maximizing vision, but must always include exercise-based fall prevention programs (FPPs) (2, 12). These FPPs consist of physical exercises to improve both strength and balance and are effective at reducing fall rates and risk of falling among older adults (2, 9, 13).

Despite that FPPs have been proven to be successful in research settings, their effectiveness in practice is limited due to significant levels of non-engagement and-adherence, and the reluctance of many older people to participate, leading to low uptake rates (8, 14). In line with this, health and social care professionals experience difficulties in recruiting and motivating older people at risk of falling to participate in FPPs. To bring FPPs to their full potential, studies investigated factors associated with engagement in fall prevention and identified, among other things, that people often have negative perceptions related to falls and aging, such as embarrassment, vulnerability, and dependence (8, 15). For instance, older people view falling as a threat to their identity and see themselves as ‘not the type to fall’ (16) and are thus not likely to take preventive measures. On the other hand, worries about falling are common among older people and how they will act on these worries seems to depend on the perceived locus of control (17). All these factors are major influencers for adults at risk of falling to engage in preventive behaviors, such as participation in FPPs (8). As motivating and engaging the target population in FPPs is of key importance to achieve a reduction in fall rates, insight is needed into how to engage community-dwelling older adults in FPPs and motivate them to participate. Therefore, this study aims to identify promising strategies that promote participation in FPPs among community-dwelling older adults at risk of falling.

## 2. Materials and methods

### 2.1. Design

This is an exploratory qualitative study on the perceptions of community-dwelling older adults on potential strategies that had been designed to promote participation in FPPs. It is part of a Dutch implementation research project: Fall pRevention ImplEmentatioN stuDy (FRIEND), which received ethical clearance from the Ethical Committee Research Healthcare Domain of the HU University of Applied Sciences, Utrecht, Netherlands (113–000-2020). To contribute to explicit and comprehensive reporting of qualitative studies, we completed the Consolidated Criteria for Reporting Qualitative Research (COREQ) checklist (Additional File 1) (18).

### 2.2. Approach

In previous studies of the FRIEND project, we identified barriers and facilitators for participation in FPPs by older adults. This was achieved by a quick literature scan, complemented with semi-structured interviews among community-dwelling older adults aged 65 years and older at risk of falling (19). Next, based on the barriers and facilitators, we designed potential strategies for stimulating the target group to participate in FPPs. This was performed by the researchers (MS, LV, LD, AR) during multiple iterative sessions, using the Intervention Mapping approach. Intervention Mapping is a planning framework that provides theory/evidence-based behavior change methods that could influence barriers and facilitators (20). These methods can be translated into practical strategies. Also, input was collected from professionals from the field: health and social care professionals, and experts in the area of implementation and working with older adults. This resulted in 14 potential strategies, of which similar ones were combined until we reached six unique potential strategies: (1) reframing; (2) informing about benefits; (3) raising awareness of risks; (4) involving social environment; (5) offering tailored intervention; (6) arranging practicalities (19) (Table 1). In the current study, these six strategies were presented to community-dwelling older adults at risk of falling. The main goal was to identify whether older adults considered these strategies as important to take into account, to promote participation in FPPs.

**Table 1:** Clarification of the six previously designed potential strategies to promote participation in FPPs among community-dwelling older adults.

Strategy	Clarification
1. Reframing	Avoid negatively connotated words such as ‘older adult’ and ‘falling’
2. Informing about benefits	Inform about benefits of participating in an FPP, such as ‘living independently at home’ or ‘staying healthy’
3. Raising awareness of risks	Inform about risk factors for falling and the negative consequences of falls
4. Involving social environment	Stimulate support from the social environment, for example, family, friends, and peers, but also involve recreational networks and organized activities in the neighborhood
5. Offering tailored intervention	Tailor the intervention to personal needs and wishes
6. Arranging practicalities	Take into account practical issues to enable participation in an FPP (e.g., accessibility, location, costs, scheduling)

### 2.3. Respondents

Respondents were selected based on convenience sampling within four districts in the region of Utrecht, Netherlands. We recruited respondents in two ways. Physical therapists involved in the FRIEND-project were asked to inquire whether their patients who met the inclusion criteria and would be interested in participating in a research interview. If they were, their contact information was sent to a researcher (MS) with the consent of the potential respondents. Also, we asked a community center to spread a study flyer among their older members. Those interested could contact the researchers by sending an e-mail with contact details. After receiving contact details, the potential respondents were called by a researcher (MS) to check whether they met the inclusion criteria. During this telephone call, no other personal information than necessary to verify eligibility was shared to prevent possible assumptions about participation in the study, relationship establishment, or other biases.

Respondents were included when they were: (1) aged 65 years and older and (2) at increased risk of falling. These criteria reflect the target group of FPPs. Increased fall risk was identified using two questions: “Have you had a fall in the past 12 months?” and “Do you experience difficulties in balance, mobility, or gait?.” Answering “yes” to at least one question indicated an increased risk of falling. This is in accordance with Dutch guidelines for fall risk screening (21). Respondents were excluded when they were not able to understand or speak the Dutch language well. When the eligible respondent was willing to participate, an information letter was sent by email or post, and the interview was scheduled at a date and time that suited the respondent. The respondents received two options for conducting the interviews: via telephone or video-conferencing. Telephone interviews were preferred by all respondents over interviews via video-conferencing since it was easier for them to use the telephone than the computer. The respondents received contact information from the researchers if any questions arose before the interview or if they decided that they eventually did not want to participate in the study.

### 2.4. Study procedures

Four female researchers (MS, LV, LD, JB) held single-time semi-structured interviews by telephone between December 2020 and March 2021. The interviews were conducted during a COVID-19 lockdown. Therefore, the respondents and researchers were both at home while conducting the interviews. At the time of the study, two researchers (LV and LD) were also involved in educational courses as lecturers. One researcher (MS) was involved in various (inter)national research projects in the health care domain. Another researcher (JB) had a profession as a senior advisor/project leader at a Dutch Centre of Expertise on Health Disparities (Pharos) and as a board member of the European Network of Intercultural Older Adult Care.

Three of the four researchers were experienced in conducting qualitative semi-structured interviews, the other researcher was new to this method. To ensure that interviews were performed similarly, the researchers met regularly to discuss how the interviews went to exchange suggestions for improvement. The interviews lasted approximately 60 minutes and were audio-recorded on an offline telephone. Afterward, the recordings were transferred to a secure online research database. No field notes were made.

The interview guide was developed iteratively by five researchers (MS, LV, LD, JB, AR). The final interview guide consisted of three parts. The questions in the interview guide were not shared with respondents to prevent bias.

First, the respondent and researcher were introduced and the research project was briefly described, after which informed consent was obtained. All respondents provided oral informed consent to proceed with the interview. Also, there was time for the respondent to ask questions, if necessary. Then, general interests were discussed, and demographic characteristics were collected (such as age, household composition, and educational level). This part was deliberately positioned at the beginning of the interview, to build rapport between the researcher and respondent.

The second part was about the respondents’ view on their neighborhood, in terms of how they moved around in their neighborhood, which local facilities they used (such as community centers, health centers, churches), where they met other people, and which type of health care professionals they visited. We decided to insert this part to gain insight into the neighborhood from the perspective of older adults and to identify specific leads on how and where to apply certain strategies. For example, when we know at what types of locations older adults meet each other, we can recommend organizing FPPs at these locations or spreading information through those particular organizations.

In the third part, the six previously designed strategies were presented in an informal manner to be understandable for the respondents. For example, to discuss the strategy ‘reframing’, we asked respondents which words they would choose in information material about FPPs aimed at people of similar age. Also, strategies were validated by asking whether a given strategy was important to consider to stimulate participation in FPPs.

The final version of the interview guide had open-ended questions and was tested by one of the researchers (MS) in a semi-structured interview with one person from the target group who met the inclusion criteria. At the end of the interview, feedback was collected on the clarity of the questions and the difficulty of answering the questions. During meetings among the researchers, the respondents’ feedback and the experiences of the researcher were discussed, which led to minor changes in the questions in the interview guide. Data from this interview was not included in the data analysis.

### 2.5. Data analysis

The audio recordings of the interviews were transcribed verbatim. Transcripts and finalized findings were not returned to respondents. Four researchers were involved in the data analysis (MS, LV, LD, AR), for which ATLAS.ti Windows (Version 22.2.4.0) was used (22).

**Figure 1:**
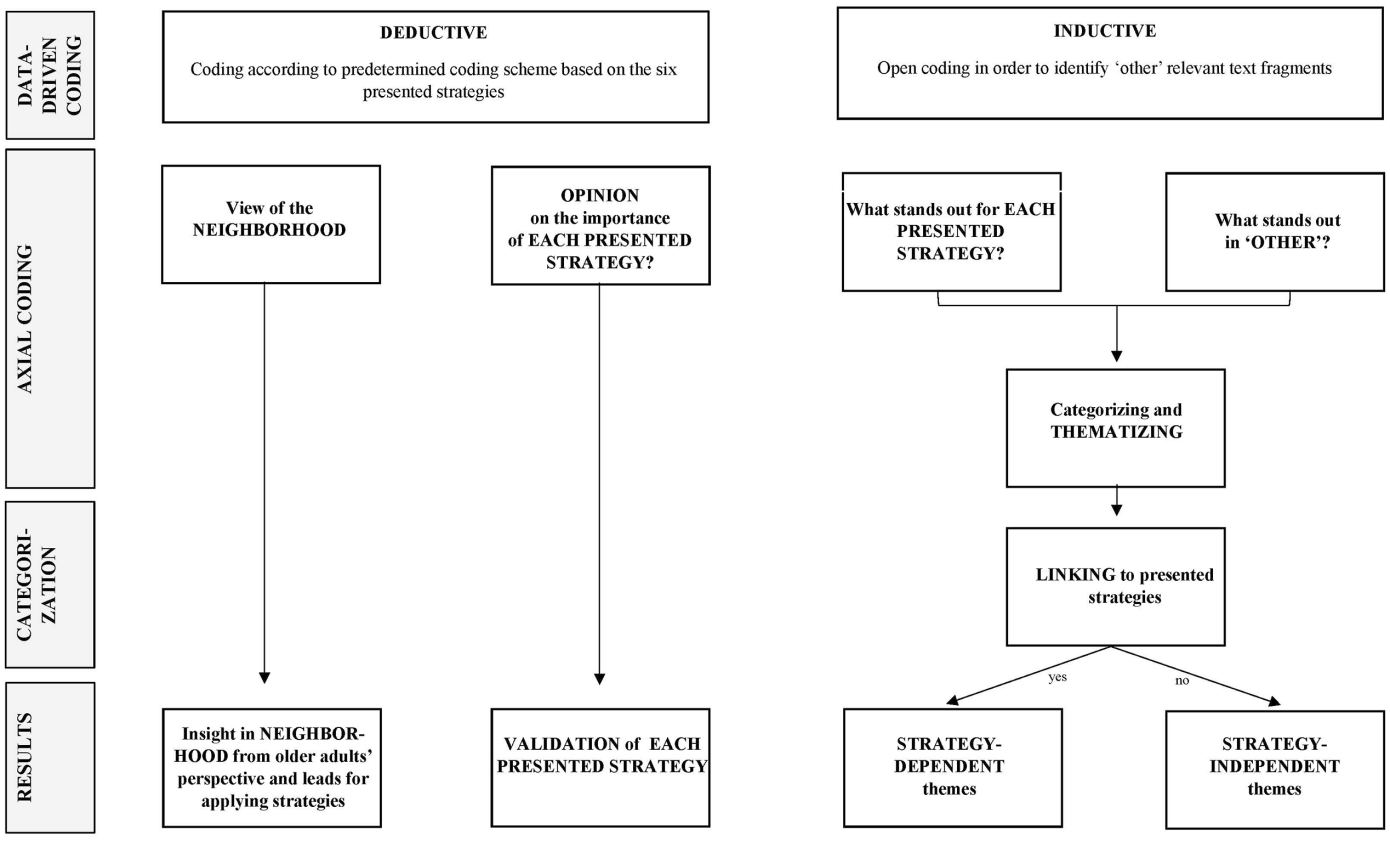
Process of the hybrid qualitative thematic analysis of the data from semi-structured interviews with community-dwelling older adults (*n* = 12) on the six presented strategies to stimulate participation in FPPs.

Data analysis was performed using a hybrid qualitative thematic methodology, combining deductive and inductive approaches (Figure 1). The first two parts of each transcript were coded by one researcher; the last part was coded separately by two researchers; the combination of researchers was different for each transcript. The researchers (MS, LV, LD, AR) independently read the appointed transcripts throughout to achieve familiarization and develop a sense of the entire dataset. Codes were derived by highlighting relevant fragments in the text that appeared to capture core thoughts or leads. The codes used the exact words of the transcript, enabling us to remain as close to the data as possible (i.e., data-driven codes) (23). After both researchers of a duo finished coding a transcript, they met to reach consensus on the codes.

The hybrid qualitative thematic analysis comprised several stages (Figure 1). During the first stage (*data-driven coding*), a coding scheme with predetermined codes based on the topics and sub-topics of the interview guide was created. The coding scheme was used for the deductive part of the analysis, and structured the coding process, ensuring that the researchers executed the coding similarly. This deductive approach enabled us to identify any leads about their neighborhood and allowed for analysis of respondents’ opinions and thoughts on the six presented strategies. The inductive analysis method implied that seemingly relevant text fragments that did not fit the scheme, were coded as ‘other’.

Next, at the second stage (*axial coding*), two researchers (MS and LV) summarized the codes of the part ‘view of the neighborhood’ (i.e., what kind of local facilities were used by the respondents in each district). Then, per strategy, two researchers (MS and LV) reviewed the codes that were derived deductively from the data, for example, specific words the respondents preferred to use in information about FPPs. Also, codes on whether the respondent thought that the strategies were indeed important to stimulate older adults to participate in an FPP were summarized. This enabled us to validate the presented strategies.

Furthermore, all researchers who were involved in the data analysis (MS, LV, LD, AR) performed an inductive analysis of the data. This was done by identifying remarkable codes within each of the six presented strategies and the ‘other’ codes, by answering the question: what stands out? An overview of remarkable codes was created per strategy and for the ‘other’ codes. Afterward, connections between the remarkable codes were drawn to draft themes.

Then, during the third stage (*categorization*), the researchers (MS, LV, LD, AR) discussed whether emerged themes could be linked to one of the six presented strategies. The categorization was final when the researchers reached consensus. This resulted in ‘strategy-dependent themes’. When themes did not fit into one strategy but emerged within multiple strategies, we categorized them as ‘strategy-independent themes’.

## 3. Results

### 3.1. Description of the final sample

In total, 20 people indicated their interest in the study. Of these, three were recruited via a community center, and 17 via physical therapists. All potential respondents were eligible for inclusion in the study. Eight potential respondents pulled out after initial contact, resulting in a sample of twelve respondents. The main reasons were either because an interview was too time-consuming, or they thought they could not contribute much because they had only experienced a few falls and therefore felt they did not have much to share.

The mean age was 77 years (range 65–86). Eleven respondents were female. Almost all respondents lived alone (*n* = 11). All respondents at least finished secondary school, and 5 of them attained a higher professional education level or university level (Table 2).

**Table 2:** Characteristics of the respondents of the semi-structured interviews.

Characteristic	Total sample (*N* = 12)
Age in years: mean ± SD (range)	77 ± 6.3 (65–68)
Gender (*n*)	Female (11)
Household composition: living alone (*n*)	11
Educational level (*n*)	
Primary school	0
Secondary school	5
Secondary vocational education	2
Higher professional education	3
University	2

SD, standard deviation.

### 3.2. View of the neighborhood

In most neighborhoods, there was some kind of social facility where older adults could meet other people. For example, churches, community centers, or activities organized in a residential complex. Respondents knew their way around in their neighborhood and traveled primarily by foot or public transport. During the daytime, they felt safe in their neighborhood, but some were hesitant to go out after dark. Several respondents experienced limitations in mobility due to a previous fall. The general practitioner and the physical therapists were among the most visited health care professionals. Other professionals included medical specialists, pharmacists, and home care nurses. The level of familiarity with fall prevention activities differed between respondents. Most were not aware of fall prevention activities; some did already participate in regular group physical exercises at a physical therapy practice or other sports facilities, and one respondent was aware of local fall prevention activities but had not participated in these before.

### 3.3. Synthesized summary

Overall, all presented strategies were recognized and supported to some extent by respondents. Results are reported per presented strategy. First, the results from the deductive analysis are described, followed by those of the inductive analysis, i.e., themes that emerged within the presented strategies (strategy-dependent themes). Then, strategy-independent themes that emerged are described (Table 3). Results are illustrated with quotes from the interviews, translated from Dutch to English.

**Table 3:** An overview of the six strategies that were presented to community-dwelling older adults, with emerged strategy-dependent themes, and strategy-independent strategies.

Presented strategies	Strategy-dependent themes that emerged^*^
1. Reframing	- ‘Aging’ and ‘falling’ are stigmatized topics - Life-course perspective on falls
2. Informing about benefits	- Credible and realistic messages - Be aware of patronizing messages - Currently incorrect beliefs about FPPs
3. Raising awareness of risks	- Scaremongering
4. Involving social environment	- Incorporating FPPs into existing local activities
5. Offering tailored interventions	
6. Arranging practicalities	
Strategy-independent themes that emerged	
	- Humor - Approach

^*^Emerging themes are themes that arose during the inductive analysis of the presented strategies and which were ‘new’ information. Blank cell = no themes emerged during inductive analysis of the presented strategy.

#### 3.3.1. Strategies and strategy-dependent themes

##### 3.3.1.1. Strategy 1: reframing

Several respondents deemed this as an important strategy. They experienced words such as ‘older adult’ or ‘seniors’ as narrow-minded terms that promote segregation. Respondents mentioned that such words should be avoided and they were asked specifically which terminology they would prefer instead. Recurring answers included words that objectively addresses the target group, such as ‘aged 65 years and older’. Some respondents mentioned that they did not have a preference for wording.

*Male, 81 years:*

*“‘Seniors’ or ‘older adult’ … there you go stigmatizing people again. There are many people over 80 years of age who are really seniors, and are really old. And there are also people over 80 years of age who are still quite vital and don't feel addressed by ‘seniors’. […] ‘Over-65s’, for example, that's a little bit more neutral.”*

###### 3.3.1.1.1. Emerging themes

One of the major themes that arose from the data within this strategy was that ‘fall’ and ‘fall prevention’ were perceived as stigmatizing topics. Talking about ‘fall prevention’ using these words was unpleasant for almost all respondents. ‘Falling’ was felt to correlate with being old and frail, and as respondents often did not see themselves as being old or at increased risk of falling, they did not recognize fall prevention as relevant to them. However, they felt that they were being stereotyped by others as such.

*Male, 81 years:*

*“To start talking about 'fall prevention'… I think that also puts a stigma on ‘falling’ because people say: “I don't fall”, but they do fall of course.”*

As an alternative framing, some respondents suggested a ‘life course perspective’ on falling, since falls occur throughout the life span, and not only at an older age.

*Female, 69 years:*

*“But shouldn’t we talk about falls befóre 65 years of age. I fell in the house, down the stairs, when I was 46 years. So anyone can always fall; you only need one unguarded moment.”*

##### 3.3.1.2. Strategy 2: informing about benefits

This strategy was perceived as important by some respondents; they mentioned that a positive message would likely be appealing and stimulating. Respondents exemplified the type of benefits that could be described, such as ‘living independently for a longer period of time’, or ‘staying fit and healthy’. Also, they talked about the possibility to share these positive messages through stories of peers, for example in magazines.

*Female, 81 years:*

*“That you can approach it from a positive angle, and not mention disadvantages. The advantages should be more on the foreground, in my opinion. And that it gives you the chance to live independently for longer. I think that's the most important thing.”*

*Male, 81 years:*

*“I notice that the whole prevention circus… that's not a priority. They [older adults] want to be able to focus their lives on other things.”*

In addition, informing about the benefits of participating in FPPs can be appealing to those who do not feel that falling and fall prevention concerns them. Communicating about matters that are relevant to them, such as being able to walk safely outside, or being active and fit, can increase the potential to reach a large proportion of the older population at risk of falling.

###### 3.3.1.2.1. Emerging themes

Some respondents emphasized that a positive tone should not be overdone, as this would jeopardize the credibility of the message. Overly positive messages can mismatch people’s expectations of life at this later stage.

*Male, 82 years:*

*“The idea that you can prevent everything… You really shouldn't hint at that too much, because I don't think any older person believes that. Besides, that conjures up an image that for all older people there would be this kind of perspective, well, that's the utmost nonsense.”*

Another remark given on informing about the benefits of fall prevention was, that older adults should not be patronized.

*Male, 82 years:*

*“Within that […] approach, you shouldn’t go too quickly in the direction of ‘that would be good for you’, because we know it ourselves. You shouldn’t, just shouldn’t do that.”*

A last theme that emerged when discussing this strategy, was that some respondents lacked information, or had incorrect beliefs about FPPs. For example, one respondent thought that participants of an FPP just practiced the act of falling, which she considered risky at an older age. These beliefs can hamper participation. Providing the right information is therefore important and individual preferences should be taken into account.

*Female, 81 years:*

*“Well, look, a course about ‘learning to fall’… I’m not in favor of that. Because you can fall wrongly at some point anyway. I mean, you can say ‘I’m learning how to fall’, but you would do that when you are 20 or 25 [years old], not when you are 60 or 70.”*

However, not all respondents felt that informing about benefits would indeed stimulate older adults to participate in FPPs. Specifically, improving fitness and health was mentioned to be less appealing to those who already feel fit and healthy.

##### 3.3.1.3. Strategy 3: raising awareness of risks

Some respondents mentioned that it was important to inform older adults about risk factors for falling and to emphasize the negative consequences of falls for health and wellbeing. One respondent explicitly mentioned that by raising awareness of risks, people might get warned that they have a risk of falling and what they can do to prevent a fall. It was not expected that this strategy would lead to participation in FPPs directly, but might stimulate cautious behavior.

*Female, 77 years:*

*“Mentioning why older adults end up in a hospital after a fall, what reasons there are for falling and why it is important to pay attention […]. There are, of course, many people with fractures and I think it is very important to mention this. ‘Watch out, there is a doorstep; watch out, you’re turning too fast or you’re not paying attention to what you are doing, which may cause you to fall’.”*

###### 3.3.1.3.1. Emerging themes

Respondents thought that within this strategy, scaremongering could lead to negative health behaviors. One respondent gave an example of a peer who did not go out of the house at all to prevent a fall. Also, some respondents indicated that part of the older population is not interested in this topic, and therefore, this strategy would probably not affect them.

*Male, 82 years:*

*“You shouldn’t scare-monger either. […]. I once had a good friend, who did allow herself to get affected by scare-mongering. She did not come out of the house at all, for the purpose of ‘prevention’.”*

*Female, 76 years:*

*“When you do not experience any problems, than you’re probably not very open to the topic. If you don’t know anything about it, than you’re not very interested.”*

##### 3.3.1.4. Strategy 4: involving social environment

Respondents mentioned that to reach the older population, it is important to promote FPPs through the social environment. More particularly, community-based social places (for leisure activity) where peers already get together, was mentioned to be a helpful way to get in contact with older adults. Some respondents indicated that these places were stimulating to start up the conversation about falls and fall prevention. Also, word of mouth in the social environment was considered to be helpful for older adults to motivate each other to participate in FPPs.

*Female, 77 years:*

*“The social environment is of great importance, because that's the best source to get contact. And that the social contacts are cheering each other on, so to speak, to participate in something.”*

*Male, 81 years:*

*“Especially the use of the social environment, of support from the environment [is important]. It could be a possibility to organize something in the neighborhood that invites residents to talk to each other, so that it really becomes a bit more of a topic of conversation as well, this ‘falling’ and ‘fall prevention’."*

Additionally, some respondents mentioned that it could be helpful to organize a local activity, such as a theatre show or a market, with a demonstration of what an FPP entails, and where people can try different elements of FPPs (e.g., exercises).

*Female, 65 years:*

*“And if the GP and other professionals involved would organize a ‘happening’ twice a year, a market for example. And that they make sure they physically challenge them, resulting in them falling or nearly falling. Or that we have to crawl underneath something… That would be appealing for me.”*

*Female, 77 years:*

*“A theatre performance seems like a great idea. I think there will be quite an interest in that. That you can show, in a very fun way, what is serious about falls, and what are the consequences of falls, but you can put that in a fun setting. I would like it very much.”*

###### 3.3.1.4.1. Emerging themes

Respondents mentioned that offering FPPs in addition to already existing physical activities in the neighborhood, such as gym classes, might help to stimulate older people to participate in FPPs.

*Female, 77 years:*

*"I am sure that if they had offered it at our gym club, I definitely would have participated.”*

##### 3.3.1.5. Strategy 5: offering tailored interventions

Respondents felt that this strategy was important to consider when stimulating older adults to participate in FPPs: professionals providing FPPs should take into account that every individual has different interests and needs to which the intervention should be tailored.

*Female, 85 years:*

*“I think it is very important. They (older adults) are all different, of course. You have to take everyone into account separately. One person can't do this and the other can't do that. One can't ride a bike and the other can't do that. You should have to take that into account a little bit.”*

##### 3.3.1.6. Strategy 6: arranging practicalities

*Female, 77 years:*

*“As it starts costing money, and a lot of money, then there is no chance people will participate. On the basis of this problem, I would like to address to the insurance companies that they must do something about it.”*

*Female, 65 years:*

*“I think especially the location, so that it is central in a neighborhood […]. Maybe you should also offer it at multiple locations then."*

Some respondents recognized the importance of this strategy. In particular, some respondents indicated that making sure that the costs associated with an FPP were covered (e.g., through healthcare insurance), would enhance their motivation to participate. Furthermore, organizing an FPP at an accessible location seemed to be essential. The location should be centrally located in the neighborhood so that it is nearby and available for all people.

Besides location, the time of day was mentioned as an important practicality. Some respondents mentioned that people in the target group may have busy schedules, for example, because of babysitting their grandchildren or social activities. However, individual differences were found to be large, making it challenging to find the right time slot.

*Female, 75 years:*

*“I think you have to give people a choice, because there are people who want it [an FPP] very early in the morning and other people want it a little later in the day. That's also very personal. So I suppose, if there are multiple times that it is organized in a day, that you have a choice. That seems important to me.”*

#### 3.3.2. Strategy-independent themes

Other strategies that emerged from the data were (a) humor and (b) personal approach.

a) Humor: humor was expected to be helpful in communicating about FPPs with older adults. Respondents assumed that humor would facilitate knowledge uptake, and decrease perceived barriers to talking about the unpleasant topic of falling.

*Female, 69 years:*

*“What also works here, I think, is laughter. There is no education as strong when it is intertwined with laughter. You absorb an awful lot of knowledge when you laugh.”*

b) Personal approach: respondents mentioned that it is important to have an actual person, preferably a health or social care professional, who initiates the contact about fall prevention, as this would stimulate them to participate in FPPs. Additionally, posters or flyers could be distributed to start the conversation about fall prevention. Whether this would also stimulate the intention to participate remained uncertain.

*Male, 81 years*

*“I can be brief about that [what the most appropriate channel is]: that has to be trough a personal approach. Because with flyers; you pick them up and then you take a look at them, but the chance that it leads to further actions is - in my case - not great. […]. I never believe that a flyer is able to get people willing to join a fall prevention course or something like that […]. Maybe it may have its use when such a flyer has been lying around for a while and they have taken it and someone comes to ask them about it, and than it is something familiar. But it won’t be enough."*

## 4. Discussion

Although there is an extensive and growing body of evidence that supports the effectiveness of FPPs to reduce fall rates in the older population (11, 12), participation rates in FPPs are low (24–26). This study provides insight into potential strategies to stimulate participation in FPPs among community-dwelling older adults. All presented strategies were recognized to some extent by the respondents as being relevant and several additional themes were identified that seem to play a major role in the stimulation and motivation of older adults to participate in FPPs.

One of the most prominent themes that emerged from the data within the strategy ‘reframing’ was that ‘old’, ‘falling’, and ‘fall prevention’ are stigmatized topics, from which older adults prefer to dissociate themselves. This leads to respondents not recognizing themselves as being ‘old’ or a ‘faller’ and therefore a lack of urge to participate in FPPs. This implies that reframing is an important strategy to promote participation in FPPs. Research has shown that older adults experience ‘falling’ as a symbol of being disabled, loss of independence, and frailty (14, 15, 27). While the strategy ‘reframing’ was primarily designed to only address negatively associated words with being old, such as ‘older adult’ or ‘seniors’, results showed that it is equally important to reframe topics regarding ‘falling’ and ‘fall prevention’. It was suggested to take a life-course perspective about falls since falls do not only occur to older adults but instead are an inevitable part of the entire lifespan. This is in accordance with the recently published report of the World Health Organization on preventing and managing falls (28). They suggest a life-course approach to fall prevention and that preventive measures should be taken from early childhood, such as encouraging regular physical activity (28).

Moreover, informing older adults on the perceived benefits of participating in FPPs could be appealing and stimulating for them, when framed positively and without mentioning it is about a program focusing on ‘fall prevention’. Many older adults do not see themselves participating in FPPs, as this relates to being ‘old’ and a ‘faller’, but do find it important to stay fit and healthy, to be able to live independently at home for as long as possible, and in good health. Therefore, older adults may be more willing to participate in exercise programs that emphasize the benefits of FPPs, such as helping to stay fit and healthy and being able to keep performing hobbies and other activities that are important to them (27, 29).

The importance of positively framed communications about falls (prevention) and age has been highlighted in many other studies as well (15, 27, 29, 30). Messages that promoted health and independence, rather than falls, were shown to be more effective to raise awareness of fall prevention among community-dwelling older adults than messages about falls and fall prevention (15, 31). However, data in the current study showed that these messages should not be overly positive, since this could reduce the credibility of the message. Also, negative messages including raising awareness of the risks of falling in advanced age have been criticized in previous research (31). A report on Reframing Aging and Ageism concluded that fear-based messages may gain attention in the short-term, but discourage engagement over a longer period of time (30). In general, message framing is one of the most researched topics in health communication, while still least understood. The effectiveness of loss- (or negative) and gain- (or positive) framed messages depends largely on the target audience and its perceptions and processing style (32). When the target audience is less familiar or feels less involved with the topic, loss-framed messages are less effective, and positive messaging may be more successful (32, 33). In the current study, results suggested that the target audience often feels that ‘fall prevention’ does not concern them, which leads to not recognizing personal relevance. Therefore, positively framed messaging may be preferred over negatively framed communication.

Furthermore, the importance of social support was validated by respondents in the current study. In particular, our respondents thought that an FPP provided by a sports club or other community-based center they already visited, would be easier to participate in. The places they mentioned were mainly churches, community centers, and group activities in residential settings. Also, respondents in this study mentioned that peers might encourage each other by discussing the topic. Previous research has also shown that social influences from families, peers, and health care professionals in the community are essential in stimulating older adults to participate in exercises or fall prevention (34, 35). Specifically, family members and friends’ concerns and their positive reinforcement influenced engagement in fall prevention (35).

In addition, personal preferences between individuals vary greatly, and this has to be taken into account as well. Respondents in the current study thought that personalizing the intervention to their needs and wishes was important, making FPPs more appealing. Other studies have shown great variation in personal factors (such as health status, physiological, and physical factors) that influence adherence to exercise programs in older adults (8, 36). This underlines the importance of tailoring the intervention to personal preferences and needs while developing strategies for increasing participation in FPPs, which is also highly recommended in the World Falls Guidelines (2).

In addition, in the current study, respondents also expressed the importance of addressing practical issues of an FPP. Especially, providers should ensure that the location of the FPP is easily accessible. Moreover, costs were experienced as a major barrier, and therefore compensation through, for example, health insurance should be considered. Such program factors (such as supervised programs, session frequency, location, fee, and intensity of the intervention) have been shown to influence older adults’ adherence rates to exercise programs in previous studies as well (8, 36, 37). Legislation for financing FPPs is currently changing in Dutch policies, making room for extra funding of FPPs.

Interestingly, two themes (personal approach and humor) emerged from the data during the discussion of multiple strategies (strategy-independent themes). The theme ‘personal approach’ emerged from the data, as respondents mentioned that information about fall prevention could be spread through different channels. Some preferred to be informed about fall prevention by professionals in the field. Results showed that the physical therapist and general practitioner were among the mostly visited health care professionals; they play an essential role in bringing up ‘fall prevention’. Additionally, flyers, posters, and advertisements could help to start the conversation about fall prevention. Previous research on recruitment strategies concluded that, for recruiting participants in research including physical exercise interventions, advertisement in local newspapers was the most effective method. Mass media or billboards were less effective, whereas interpersonal contact such as word of mouth was a more successful strategy for recruitment (38, 39). Furthermore, some respondents mentioned that using humorous communication would help to open up the dialogue about ‘falling’ and ‘fall prevention’ easier.

### 4.1. Strengths and limitations

A major strength of this study is that we consulted people from the target group themselves. The end-users are the experts in their particular area, and they are the people who need to but often fail to participate in FPPs. Participatory approaches with people who are affected by the decisions to be made, are increasingly recognized to be fundamental and have been shown to impact both the rigor and relevance of research (40). Furthermore, we performed qualitative thematic analysis using a hybrid approach, allowing us to both deductively test whether the predetermined strategies were experienced as important by the target population, and to inductively discover underlying and new themes.

There are also limitations to this study. First, we experienced difficulties in recruiting respondents. Our primary recruitment strategy was to find eligible respondents through health care professionals, but a COVID-19 lockdown with restrictions on interpersonal contacts might have resulted in hesitance to participate in research projects. Additionally, we primarily attempted to recruit a heterogeneous sample (i.e., people with various backgrounds, educational levels, and socioeconomic statuses), because it is known that such demographic factors are associated with fall rates and participation rates in FPPs (8, 41). However, due to the experienced difficulties with including enough respondents, we were forced to change our recruitment strategy and use a convenience sampling method to reach eligible respondents. This led to a relatively homogeneous (in terms of gender, household composition, and educational level) and small sample. However, we primarily aimed at exploring opinions and thoughts on the strategies. For that purpose, respondents in the sample generated enough data to help us unfold a new and rich understanding of phenomena under the presented strategies and other themes. This has been achieved by using open-ended questions in our semi-structured interviews. Second, the interviews were conducted by telephone. Although there is no evidence that interviews by telephone are of lower quality than face-to-face interviews (42), it did result in limited interaction, loss of contextual and nonverbal data, and compromised rapport. Telephone interviews also have several advantages, such as that respondents might feel more relaxed, leading them to reveal more sensitive information (39). Third, during the interviews, no field notes were taken and no member checking was performed. The use of multiple methods of data sources in qualitative research, such as transcripts and field notes, is referred to as ‘method triangulation’, enabling both to validate and develop a comprehensive understanding of the findings. In this study, investigator triangulation was iteratively performed during data analysis, to ensure thorough insights into strategies to stimulate participation in FPPs among older adults (43).

### 4.2. Future considerations

Currently, several strategies are further developed to be applied and evaluated for effectiveness in multiple field labs in a central Dutch region within the larger FRIEND project. The main focus of this follow-up research is to examine the effectiveness of one single strategy to improve intention to participate in FPPs among community-dwelling older adults at risk of falling. For this purpose, we chose the strategy that was most extensively discussed during the interviews (reframing), and within which highly remarkable themes derived. We will present two different messages about participating in FPPs to older adults at risk of falling; one with a positively reframed message and one with a neutral message. This may help to gain further insight into the effectiveness and working mechanisms of these strategies and enable us to design practical recommendations within the strategies, which health and social care professionals can apply to improve participation rates in FPPs. Additionally, in this follow-up research, we aim to recruit a large and heterogenous sample, enabling us to include demographic factors in the analyses as well, resulting in more understanding of the effectiveness of the strategy across different subgroups within the older population.

Furthermore, benefits from an exercise-based FPP do not only depend on participation and adherence but also the continuation of physical activity afterwards (8). Community-based exercise activities for older adults seem to have longer-term adherence rates, and several studies recommend health care providers to consider directing older adults to these types of exercise programs, to increase sustained physical activity (44, 45). However, less is known about different services (e.g., in the social care domain) that may be effective in promoting attendance and adherence to physical activities in the community.

## 5. Conclusion

In conclusion, it is crucial to understand which strategies may be effective to improve the low engagement and participation rates of FPPs among community-dwelling older adults at risk of falling. Overall, a personalized approach, informing about benefits, and framed in positive terms, may help promote the participation of FPPs among older adults. Future research should focus on further developing and implementing the strategies that influence older adults’ participation in FPPs. This could result in practical recommendations and help professionals in the community to promote older adults’ participation in FPPs. Next, research should be performed on establishing and implementing referral pathways after participation in an FPP, to retain older adults in regular sports and physical recreation activities in the community, and to improve long-term health benefits.

## Data availability statement

The datasets presented in this article are not readily available because it is a qualitative study; we drew conclusions based on interviews and the transcripts are not available to review due to privacy regulations. Requests to access the datasets should be directed to MS, meike.vanscherpenseel@hu.nl.

## Ethics statement

The studies involving human participants were reviewed and approved by Ethical Committee Research Healthcare Domain of the HU University of Applied Sciences Utrecht, Utrecht, Netherlands (113-000-2020). The patients/participants provided their written informed consent to participate in this study.

## Author contributions

MS, LV, LD, SV, and AR contributed to the design of the study, and conceptualized the approach for this study. MS, LV, LD, and AR performed the data collection and data analysis. MS wrote the first draft of the manuscript and led the manuscript writing. LV, AR, and SV wrote substantial parts of the final manuscript. LD reviewed the text in detail. All authors contributed to the article and approved the submitted version.

## FRIEND research group

Meike van Scherpenseel, Research Group Innovation of Human Movement Care, Research Center for Healthy and Sustainable Living, HU University of Applied Sciences Utrecht, Utrecht, Netherlands. Lidia van Veenendaal, Research group Proactive Care for Older Adult People Living at Home, Research Center for Healthy and Sustainable Living, HU University of Applied Sciences Utrecht, Utrecht, Netherlands and Bachelor Nursing Studies, Institute for Nursing Studies, HU University of Applied Sciences Utrecht, Utrecht, Netherlands. Lennie Donné, Research group Innovation in Healthcare Processes in Pharmacology, Research Center for Healthy and Sustainable Living, HU University of Applied Sciences Utrecht, Utrecht, Netherlands and Program group Persuasive Communication, department of Communication Science, University of Amsterdam, Amsterdam, Netherlands. Saskia te Velde, Research Group Innovation of Human Movement Care, Research Center for Healthy and Sustainable Living, HU University of Applied Sciences Utrecht, Utrecht, Netherlands. Amber Ronteltap, Research Group Innovation of Human Movement Care, Research Center for Healthy and Sustainable Living, HU University of Applied Sciences Utrecht, Utrecht, Netherlands. Cindy Veenhof, Research Group Innovation of Human Movement Care, Research Center for Healthy and Sustainable Living, HU University of Applied Sciences Utrecht, Utrecht, Netherlands and Department of Rehabilitation, Physiotherapy Science and Sport, University Medical Center Utrecht, Utrecht University, Utrecht, Netherlands. Marielle Emmelot-Vonk, Department of Geriatrics, University Medical Center Utrecht, Utrecht, Netherlands. Di-Janne Barten, Research Group Innovation of Human Movement Care, Research Center for Healthy and Sustainable Living, HU University of Applied Sciences Utrecht, Utrecht, Netherlands. Nienke Bleijenberg, Research group Proactive Care for Older Adult People Living at Home, Research Center for Healthy and Sustainable Living, HU University of Applied Sciences Utrecht, Utrecht, Netherlands. Rixt Zuidema, Research group Proactive Care for Older Adult People Living at Home, Research Center for Healthy and Sustainable Living, HU University of Applied Sciences Utrecht, Utrecht, Netherlands. Elise Volk, Research Group Innovation of Human Movement Care, Research Center for Healthy and Sustainable Living, HU University of Applied Sciences Utrecht, Utrecht, Netherlands

## Funding

This research was co-funded by Regieorgaan SIA, part of the Netherlands Organization for Scientific Research (NWO). The funder had no role in the conception and design of this study, data collection, data analysis, interpretation, or the writing of this manuscript (grant number: RAAK.PRO03.099).

## Conflict of interest

The authors declare that the research was conducted in the absence of any commercial or financial relationships that could be construed as a potential conflict of interest.

## Publisher’s note

All claims expressed in this article are solely those of the authors and do not necessarily represent those of their affiliated organizations, or those of the publisher, the editors and the reviewers. Any product that may be evaluated in this article, or claim that may be made by its manufacturer, is not guaranteed or endorsed by the publisher.
